# Robust reconfigurable radiofrequency photonic filters based on a single silicon in-phase/quadrature modulator

**DOI:** 10.1515/nanoph-2023-0459

**Published:** 2023-10-25

**Authors:** Hengsong Yue, Tao Chu

**Affiliations:** College of Information Science and Electronic Engineering, Zhejiang University, Hangzhou 310027, China

**Keywords:** robust radiofrequency filters, radiofrequency photonic filters, silicon in-phase/quadrature modulator, integrated microwave photonics, silicon photonics

## Abstract

Combining integrated photonics and radiofrequency (RF) signals in the optical domain can help overcome the limitations of traditional RF systems. However, it is challenging to achieve environmentally insensitive filtering in wireless communications using integration schemes. In this report, the performance of robust RF filters based on a single silicon in-phase/quadrature modulator with significantly improved temperature and optical carrier wavelength sensitivities, which were suppressed by more than three orders of magnitude compared with those of silicon resonators, was experimentally evaluated. Upconversion and the processing of signals were simultaneously realized on the modulator by setting the relative phases of the arms and the bias voltages. Moreover, the filters can be reconfigured as low-pass, high-pass, band-pass, or band-stop filters. From 25 to 75 °C, the center frequency variation was within 0.2 GHz. From 1500 to 1600 nm, the center frequency variation was within 2 GHz. The proposed scheme allows for filtering and reconfiguration without the use of optical processing modules such as resonators or delay lines, thus providing a novel approach to signal processing and a new robust filter for scenarios with dynamic environments.

## 1 Introduction

The advancement of modern communication and increasing demand for bandwidth led to bottlenecks in traditional radiofrequency (RF) systems due to the inherent limitations of electronic devices [1]. The implementation of RF systems on integrated photonic platforms significantly improves the system bandwidth, tunability, reliability, and stability, and reduces the system footprint [2–5]. Moreover, integrated photonic platforms introduce new technological tools to RF systems [6] such as Kerr microcombs [7, 8] and photonic–phononic emitter–receiver (PPER) devices [9]. In RF photonic filtering systems, integrated photonic devices such as ring resonators [10, 11], delay lines [12], and stimulated Brillouin scattering waveguides [13] are used as optical modules to process signals upconverted from radio to optical frequencies. However, these integrated RF photonic filters are not robust against temperature and optical carrier wavelength variations, which may lead to frequency drift [14]. Modern wireless communication, avionics applications, and electronic warfare have demonstrated the demand for robust filters that can retain the desired signals and suppress unwanted signals in dynamic environments [15, 16]. Nevertheless, it is difficult to avoid frequency drift using current integration schemes due to the temperature sensitivity of optical processing modules and the wavelength instability of optical carriers. This is because the filtering RF frequency is determined by the relative light wavelength of the optical processing module and carrier. For example, the thermally induced resonance shift of a silicon ring resonator is equal to 13.75 GHz/°C [17], and the optical carrier wavelength drift of the laser source causes an equal amount of RF frequency drift in integrated RF photonic filters [18].

Several approaches can be implemented to suppress the RF frequency drift of integrated RF photonic filters. Theoretically, the thermally induced resonance shift can be partly suppressed using low-thermo-optic coefficient materials. The thermally induced resonance shift of a silicon nitride resonator can reach 2 GHz/°C [19], which is similar to silica planar light-wave circuit micro-ring resonators [7]. The temperature control system can suppress the temperature fluctuations of the chip. Moreover, feedback schemes can stabilize the optical carrier wavelength of the laser [20, 21] and follow the frequency variation of the system [22]. However, these schemes increase the overall cost and power consumption, and introduce additional complexity to the system. Furthermore, residual frequency drift cannot be completely prevented.

In this study, we demonstrated reconfigurable RF photonic filters that are robust against temperature and optical carrier wavelength variations based on a single silicon in-phase/quadrature (IQ) modulator. The RF photonic filters exhibit adequate tunability and can be reconfigured between low-pass, high-pass, band-pass, and band-stop filtering. We verified that a conventional silicon IQ modulator can perform signal filtering while upconverting signals from radio to optical frequencies without the use of optical processing modules that are generally required in current RF photonic filtering systems. The filtering functions are achieved by appropriately setting the relative phases of the arms inside the IQ modulator and the PN junction bias voltages of two child Mach–Zehnder modulators (MZMs). This is based on a novel signal processing mechanism by which the frequency response of the two child MZMs can be tuned by changing the PN junction bias voltage, and the frequency response of the IQ modulator is the superimposing of the response of the two child MZMs at a specific ratio. Thus, the system architecture is simplified because the optical processing modules were omitted, and the robustness was significantly improved because the silicon IQ modulator had equal arm lengths. The suppression of the temperature and optical carrier wavelength sensitivities were experimentally verified to be more than three orders of magnitude lower than those of current silicon photonic integration systems. Considering the abovementioned characteristics and simple structure, integrated RF photonic filters allow for the realization of practical applications, especially in wireless communication and aerospace applications, among other domains.

## 2 Operating principle

**Figure 1:**
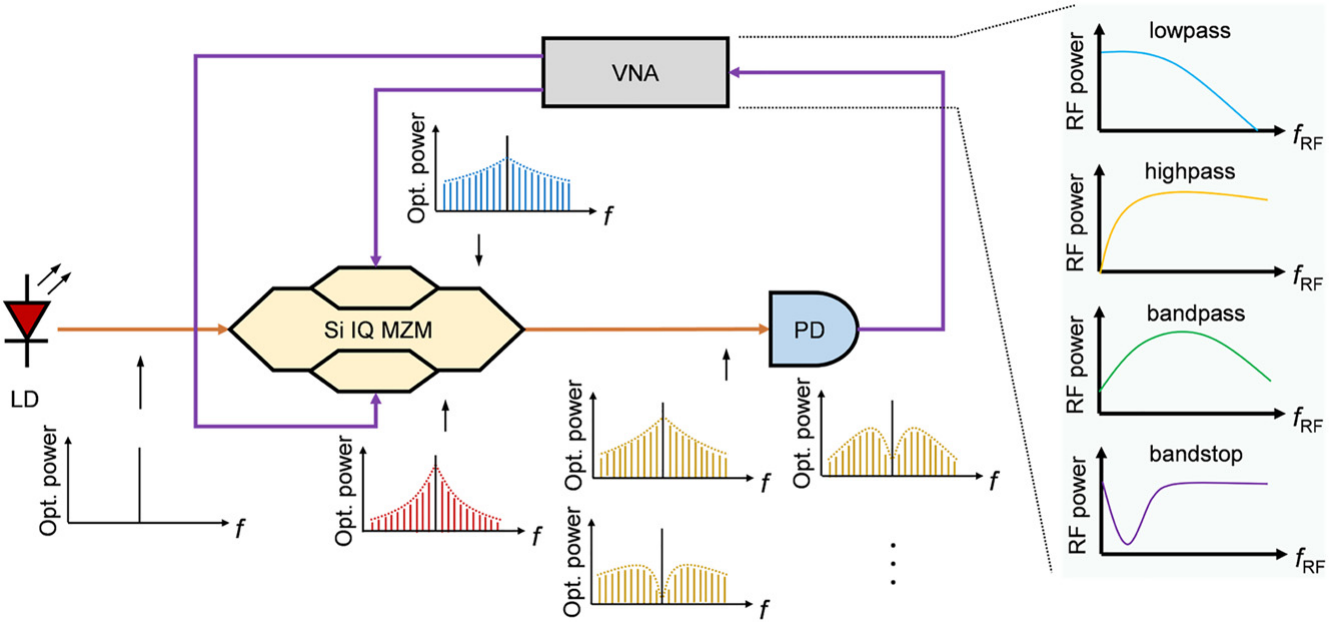
Schematic of the silicon IQ modulator-based RF photonic filters. The RF photonic filters are reconfigurable as low-pass, high-pass, band-pass, or band-stop filters by tuning the phases in the IQ modulator and controlling the PN junction bias voltages of the two child MZMs.

Figure 1 presents the conceptual setup of silicon IQ modulator-based RF photonic filters. The source of the optical carrier was a continuous-wave (CW) laser, which was then coupled to a silicon IQ modulator. The two child MZMs of the IQ modulator were driven by two identical RF signals generated by a vector network analyzer (VNA). By controlling the PN junction bias voltages of the two child MZMs, the generated amplitudes of the optical sidebands can achieve different frequency responses. Furthermore, by tuning the relative phases of the four modulating arms inside the silicon IQ modulator, the interference of the optical sidebands can exhibit various frequency-dependent responses, as shown in Figure 1. The output of the IQ modulator was fed into a photodetector (PD) for conversion into an RF signal. The beating of the interference results and the optical carrier on the PD introduces the relative phase information between them. Thus, RF photonic filters can be reconfigured as low-pass, high-pass, band-pass, or band-stop filters using only lasers, modulators, and detectors. Moreover, only one laser, modulator, and PD are required in a conceptual setup. The details of the experimental setup are presented in the Methods section.

The two child MZMs were series push-pull (SPP) traveling-wave MZMs with 20 Ω on-chip terminators to exploit the bandwidth peaking effect [23]. The child and parent MZMs had two arms of equal length and used thermo-optic tuners to set the operating points. The detailed device structure is reported in the Methods section. Assuming small-signal modulation, the two identical RF signals used to drive the two child MZMs can be denoted by *V*_RF_ sin(*ω*_RF_*t*), where *V*_RF_ and *ω*_RF_ are the magnitude and angular frequency of the RF signal, respectively. The output of the silicon IQ modulator can be expressed as follows:

(1) $$\begin{array}{ll} E_{\text{out}}\left(t\right) & =\frac{E_{0}\text{exp}\left(j\omega_{\text{c}}t\right)}{2}\text{exp}\left(j\frac{\varphi_{1}}{2}\right)\\ & \;\times \left\{\begin{array}{c} -J_{1}\left(m_{1}\right)\text{exp}\left(-j\omega_{\text{RF}}t\right)\left[-j\text{sin}\left(\frac{\varphi_{1}}{2}\right)\right]\\ +J_{0}\left(m_{1}\right)\text{cos}\left(\frac{\varphi_{1}}{2}\right)\\ +J_{1}\left(m_{1}\right)\text{exp}\left(j\omega_{\text{RF}}t\right)\left[-j\text{sin}\left(\frac{\varphi_{1}}{2}\right)\right] \end{array}\right\}\\ & \;+\frac{E_{0}\text{exp}\left(j\omega_{\text{c}}t\right)}{2}\text{exp}\left(j\frac{\varphi_{2}}{2}\right)\text{exp}\left(j\varphi_{3}\right)\\ & \;\times \left\{\begin{array}{c} -J_{1}\left(m_{2}\right)\text{exp}\left(-j\omega_{\text{RF}}t\right)\left[-j\text{sin}\left(\frac{\varphi_{2}}{2}\right)\right]\\ +J_{0}\left(m_{2}\right)\text{cos}\left(\frac{\varphi_{2}}{2}\right)\\ +J_{1}\left(m_{2}\right)\text{exp}\left(j\omega_{\text{RF}}t\right)\left[-j\text{sin}\left(\frac{\varphi_{2}}{2}\right)\right] \end{array}\right\} \end{array}$$

where *E*_0_ and *ω*_*c*_ are the amplitude and angular frequency of the optical carrier, respectively; *J*_*n*_ is the nth-order Bessel function of the first kind; *φ*_1_ and *φ*_2_ are the phase differences of the two arms of the two child MZMs; and *φ*_3_ is the phase difference between the two arms of the parent MZM. Assuming *V*_*π*1_ and *V*_*π*2_ are the half-wave voltages, *m*_1_ = *πV*_RF_/2*V*_*π*1_ and *m*_2_ = *πV*_RF_/2*V*_*π*2_ are the modulation indices of the two child MZMs. The half-wave voltages and modulation indices of the two child MZMs are frequency dependent, given that the response of the MZM varies with frequency. The frequency response curve can be obtained by calculating the normalized reciprocal of the half-wave voltage in decibels [24]. The output of the silicon IQ modulator was injected into a PD. Neglecting the frequency-doubling and direct current (DC) terms, the photocurrent can be expressed as follows:

(2) $$\begin{array}{ll} i_{\text{PD}}\left(t\right) & \propto {\left|E_{0}\right|}^{2}\text{sin}\left(\omega_{\text{RF}}t\right)\\ & \;\times \left\{\begin{array}{c} J_{1}\left(m_{1}\right)\text{sin}\left(\frac{\varphi_{1}}{2}\right)\left[\begin{array}{c} \cos\left(\frac{\varphi_{2}}{2}\right)\text{cos}\left(\varphi_{3}+\frac{\varphi_{2}}{2}-\frac{\varphi_{1}}{2}\right)\\ +\text{cos}\left(\frac{\varphi_{1}}{2}\right) \end{array}\right]\\ +J_{1}\left(m_{2}\right)\text{sin}\left(\frac{\varphi_{2}}{2}\right)\left[\begin{array}{c} \cos\left(\frac{\varphi_{1}}{2}\right)\text{cos}\left(\varphi_{3}+\frac{\varphi_{2}}{2}-\frac{\varphi_{1}}{2}\right)\\ +\text{cos}\left(\frac{\varphi_{2}}{2}\right) \end{array}\right] \end{array}\right\} \end{array}$$

where the zeroth order Bessel function of the first kind is set to 1 because small-signal modulation is assumed (see Supplementary Material, Section I for details).

**Figure 2:**
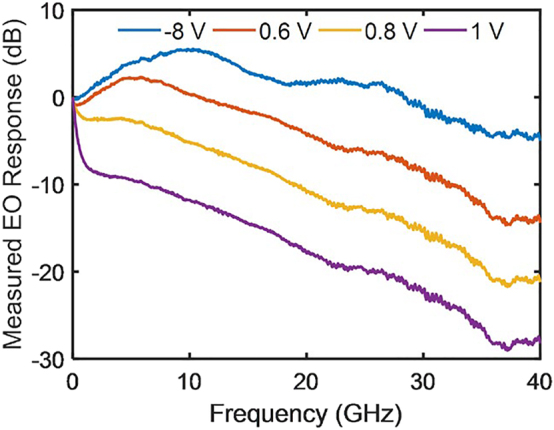
EO response of the child MZM for different bias voltages.

**Figure 3:**
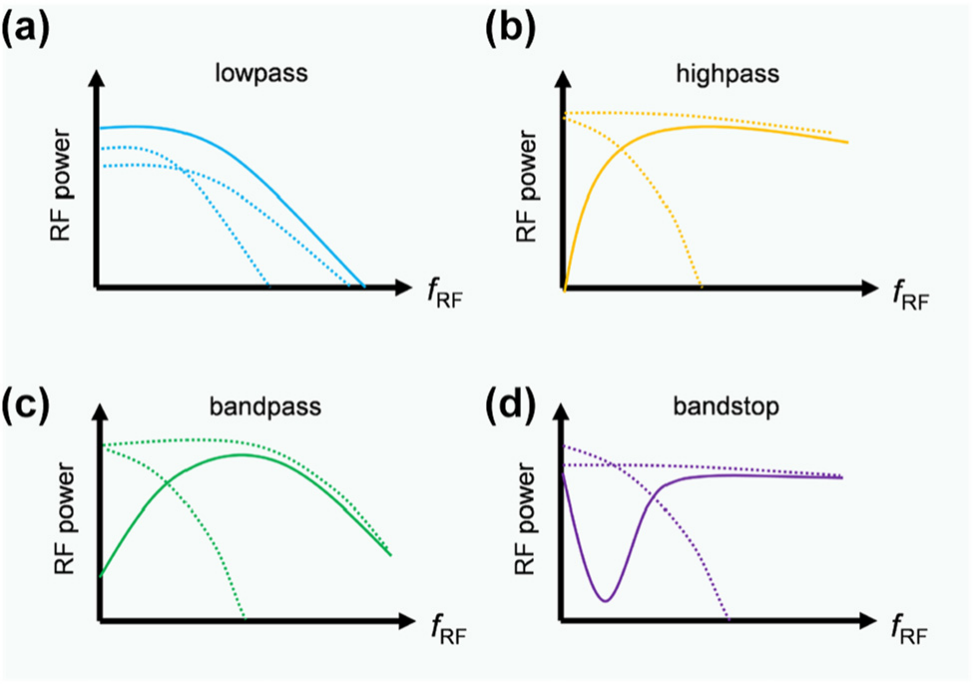
Implementation of the four filters. The dotted lines represent *J*_1_(*m*_1_) and *J*_1_(*m*_2_) with corresponding coefficients, and the solid lines represent the result of their addition (low-pass filter) or subtraction (high-pass, band-pass, and band-stop filters). (a) Low-pass filter. (b) High-pass filter. (c) Band-pass filter. (d) Band-stop filter.

In this equation, *J*_1_(*m*_1_) and *J*_1_(*m*_2_) are regarded as variables, and the ratio of their coefficients can be set to any value by adjusting *φ*_1_, *φ*_2_, and *φ*_3_. Considering that the microwave attenuation of the traveling-wave electrode is influenced by the bias voltage of the PN junction, the frequency-dependent modulation indices *m*_1_ and *m*_2_ can be tuned by changing the bias voltages of the PN junctions, which are reflected in the measured electro-optical (EO) response of the child MZM, as shown in Figure 2. Thus, reconfigurable modulator-based RF photonic filters with low-pass, high-pass, band-pass, and band-stop functionality can be implemented by appropriately setting the PN junction bias voltage of the two child MZMs and the ratio of the coefficients of *J*_1_(*m*_1_) and *J*_1_(*m*_2_). It is worth noting that the bias voltage is carefully chosen to demonstrate the prominent variations in the electro-optic response curve. The inconsistency in the chosen bias voltages can be attributed to the inherent nonlinearity of the PN junction. When the PN junction is forward biased, the forward bias current results in a significant change in the carrier density within the optical waveguide when compared to the MZM operating under reverse bias conditions. The implementation of the four filters is illustrated in Figure 3. The low-pass filtering function can be implemented by adding *J*_1_(*m*_1_) and *J*_1_(*m*_2_) with the corresponding coefficients. The high-pass, band-pass, and band-stop filtering functions can be implemented by subtracting *J*_1_(*m*_1_) and *J*_1_(*m*_2_) with the corresponding coefficients.

The filter is influenced by the microwave refractive index of the traveling-wave electrodes of the two child MZMs, which varies with their PN junction bias voltages, thus leading to a change in the EO group index matching. This mainly influences band-stop filtering because *J*_1_(*m*_1_) and *J*_1_(*m*_2_) cannot completely cancel each other at the corresponding frequency. Changing the relative electrical delay of the two driving RF signals can compensate for this effect, thus enabling a high peak rejection. In addition, ideal high-pass filtering cannot be achieved due to the limited bandwidth of the MZM and PD. Therefore, we focused on filtering functions at low frequencies that are not influenced by the limited bandwidth.

The aforementioned filtering functions are based on a single silicon IQ modulator, which simultaneously realized modulation and filtering functions, thereby obviating the need for an optical processing module. The silicon IQ modulator consisted of three Mach–Zehnder interferometers and utilized thermo-optic tuners to set operating points. Therefore, it is simple to maintain the relative phase of the inside arms while the optical carrier wavelength and temperature fluctuated [25]. Consequently, the proposed silicon IQ modulator-based RF photonic filters are potentially insensitive to the optical carrier wavelength and temperature.

## 3 Experimental results

### 3.1 Filter tunability and reconfigurability

By exploiting the wide tuning frequency response of the silicon modulator and the characteristics of the IQ modulator in which *J*_1_(*m*_1_) and *J*_1_(*m*_2_) can be combined in any ratio, the IQ modulator-based RF photonic filter exhibited excellent tunability and reconfigurability. In this study, we demonstrated the tuning characteristics of its center frequency and 3 dB bandwidth, in addition to the reconfiguration of its filtering functions.

**Figure 4:**
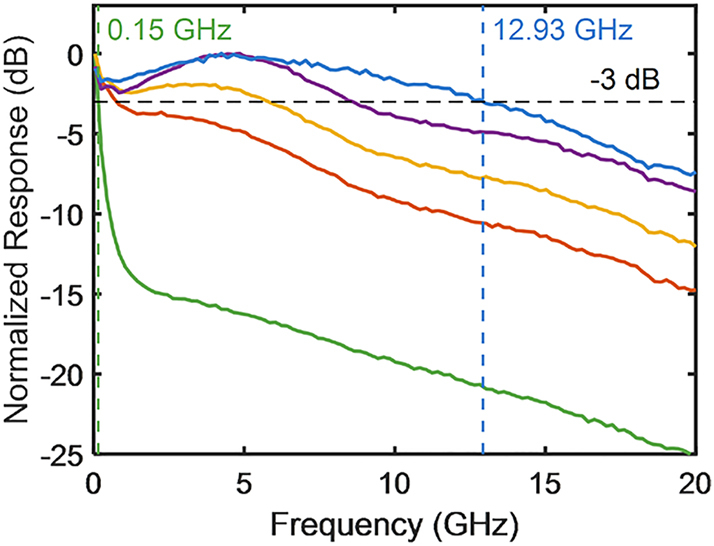
Frequency response of silicon IQ modulator-based RF photonic filters as low-pass filters. The wavelength of the laser source was 1550 nm and all frequency responses were normalized to 0 dB.

Figure 4 illustrates the response of the low-pass filters with respect to bandwidth variations. Figure 2 reveals that low-pass filters can be implemented by applying a forward bias to the modulator. However, due to the bandwidth peaking effect, which results in a reduction at low frequencies, the bandwidth of the low-pass filters implemented in this manner is limited. Setting one child MZM as reverse-biased or zero-biased and the other as forward-biased compensates for this effect and allows for a wide bandwidth. Moreover, the bandwidth can be tuned from 0.15 to 12.93 GHz.

**Figure 5:**
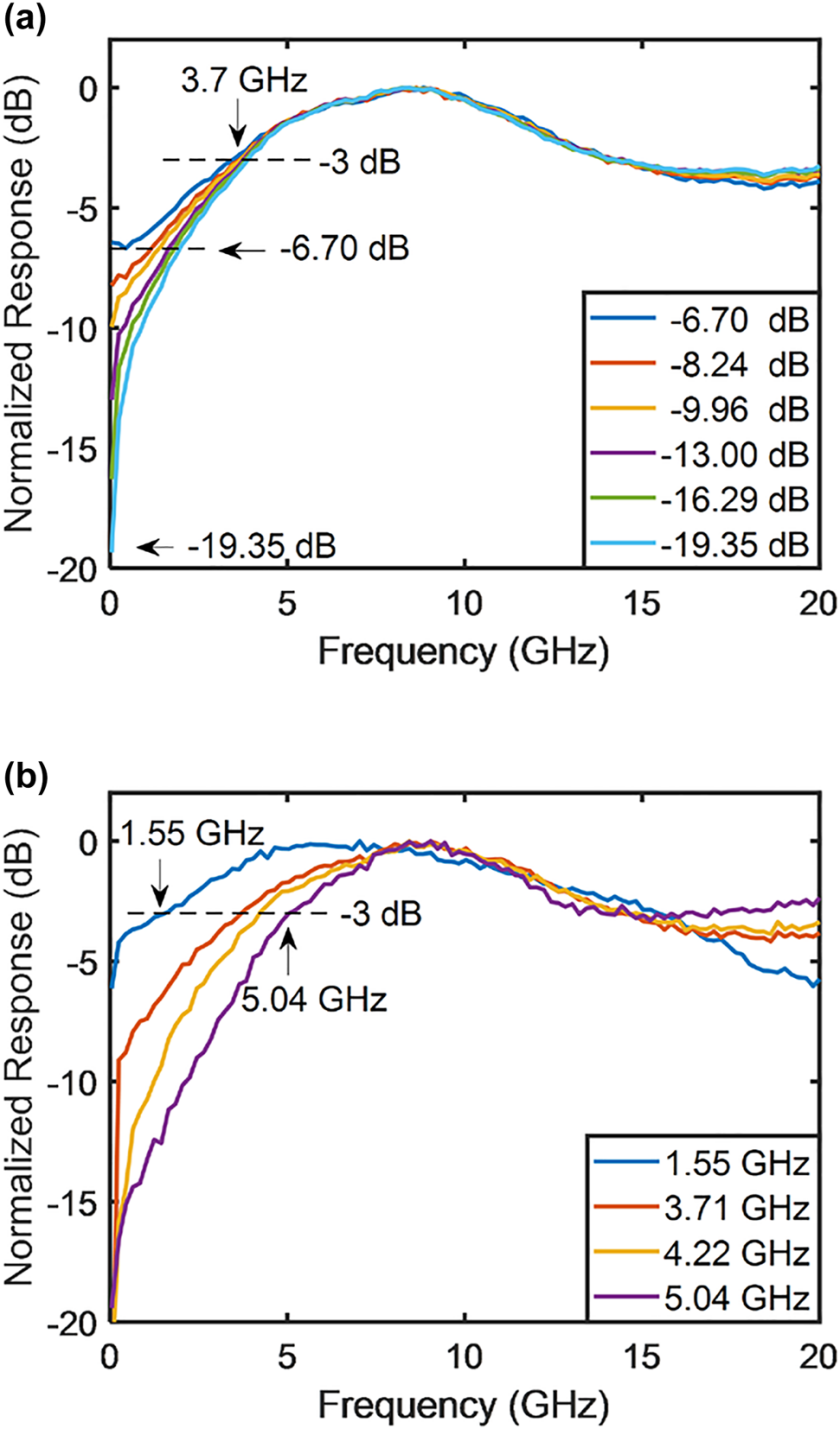
Frequency response of silicon IQ modulator-based RF photonic filters as high-pass filters. The cutoff frequency and filter depth could be tuned. All frequency responses were normalized to 0 dB. (a) Tunability of the suppression ratio. (b) Tunability of the cutoff frequency.

The tunability of the high-pass filtering function of the RF photonic filters is shown in Figure 5. When the two child MZMs had different bias voltages, the low-frequency responses of the filters could be suppressed by subtracting their Bessel functions at an appropriate coefficient ratio. Figure 5(a) illustrates the tunability of the suppression ratio. One child MZM was reverse-biased at 5 V and the other was forward-biased at 0.8 V. The high-frequency response and cutoff frequency of the high-pass filters were almost unchanged because the response of the forward-biased modulator was rapidly attenuated with an increase in frequency. The suppression ratio was tunable within a range of 6.70–19.35 dB while maintaining the cutoff frequency at 3.7 GHz. Figure 5(b) reveals that the cutoff frequency, which was mainly determined by the relative attenuation rates of *J*_1_(*m*_1_) and *J*_1_(*m*_2_) with respect to frequency, could be tuned from 1.55 to 5.04 GHz. There was no deliberate intention behind choosing those specific frequencies. The tuning process resulted in the filter operating at those frequencies coincidentally. The attenuation of the second half of each curve was caused by the limited bandwidths of the MZM and PD.

**Figure 6:**
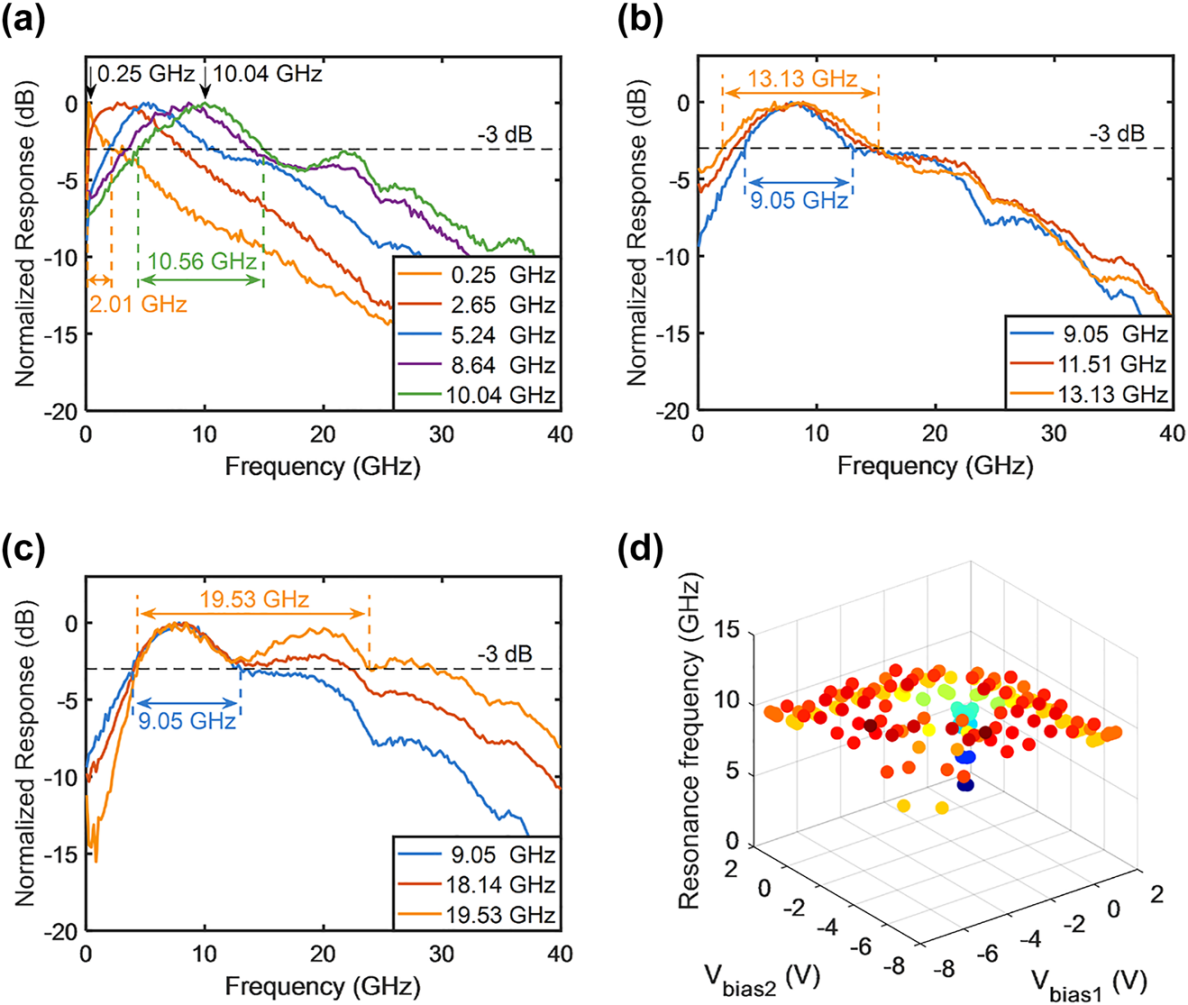
Frequency response of silicon IQ modulator-based RF photonic filters as band-pass filters. The frequency and 3 dB bandwidth of the band-pass filters could be tuned. All frequency responses were normalized to 0 dB. (a) Tunability of the frequency. (b) Tunability of the bandwidth on both sides. (c) Tunability of the bandwidth on one side. (d) Tunability of the resonance.

Figure 6 illustrates the band-pass filtering function of the RF photonic filters. As shown in Figure 2, although band-pass filters can be implemented by making the modulator operate in the reverse bias state, they exhibit limited frequency and bandwidth tunability due to the bandwidth peaking effect. However, when one child MZM is forward-biased to suppress or compensate for the low-frequency response of the other child MZM, the tuning of the frequency and bandwidth can be achieved. Changing the bias voltages can further extend the tunability. Figure 6(a) reveals that the frequency was tunable from 0.25 to 10.04 GHz, whereas the bandwidth ranged from 2.01 to 10.56 GHz. Figure 6(b) illustrates the tunability of the bandwidth, which increased on both sides. Similarly, Figure 6(c) illustrates that the bandwidth could be increased on one side from 9.05 to 19.53 GHz. Figure 6(d) showcases the tunability of resonance, calculated based on the measured EO response data of the child MZM under different bias voltages, with a partial display in Figure 2. The calculation reveals that resonance can be tuned within the range of 0.4–13.4 GHz, which aligns with the experimental results presented in Figure 6(a). The slight discrepancies can be attributed to variations in performance between the two child MZMs and the exploration of extreme conditions in the calculations, which are challenging to achieve in experimental measurements.

It should be noted that when the two child MZMs have different bias voltages, band-stop filters can be implemented by setting the coefficient ratio such that the Bessel functions of the two child MZMs are mutually eliminated at the corresponding frequencies. The change in the EO group index matching limits the suppression ratio, thus making it difficult to implement bandstop filtering at high frequencies. However, RF photonic filters exhibit sufficient tunability at low frequencies.

**Figure 7:**
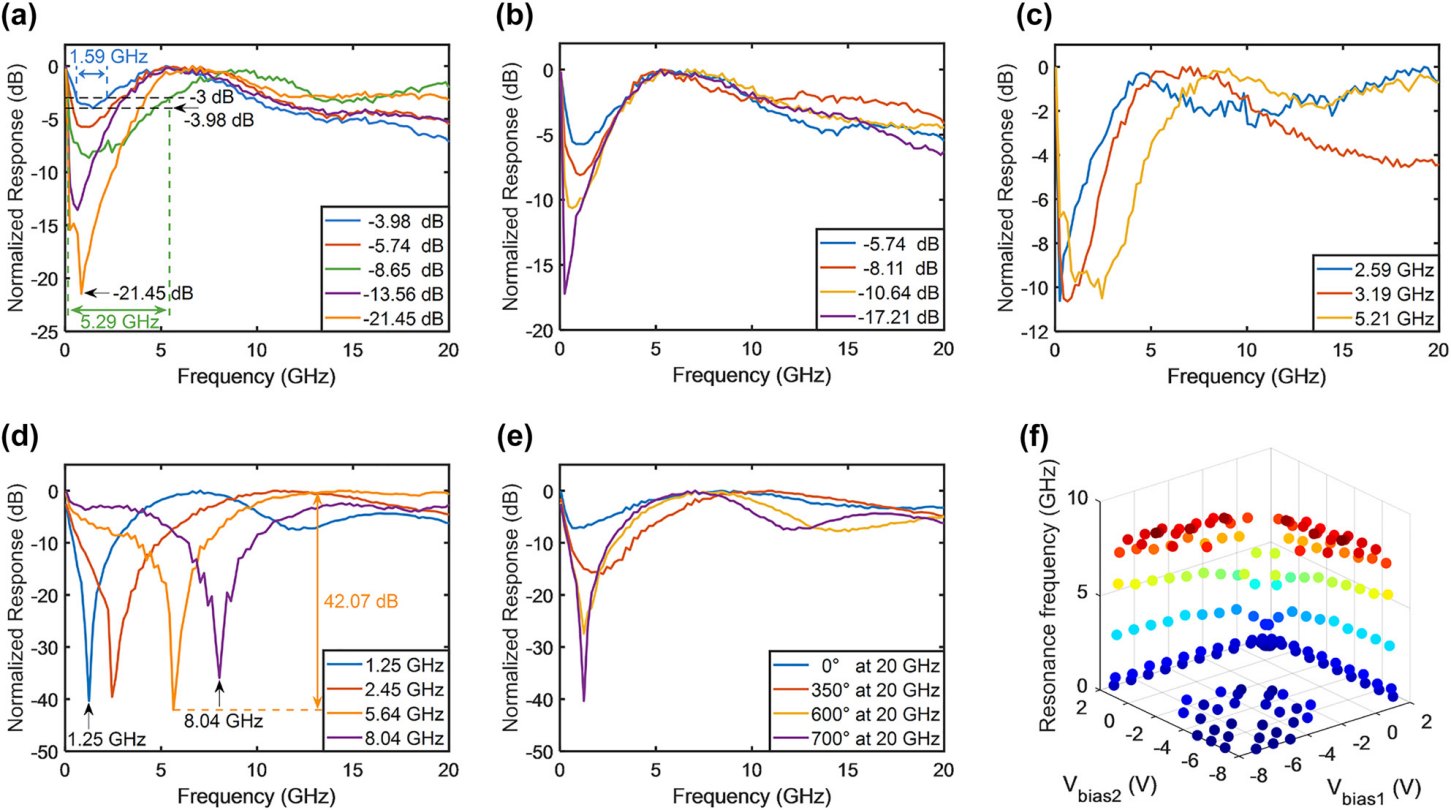
Frequency response of silicon IQ modulator-based RF photonic filters as band-stop filters. The suppression ratio and bandwidth of band-stop filters can be tuned. Electrical delay can be used to compensate for the limited suppression ratio, which is caused by the difference in the EO group index matching between the two child MZMs. All frequency responses were normalized to 0 dB. (a) Preliminary demonstration of band-stop filters. (b) Tunability of the suppression ratio while maintaining the bandwidth constant. (c) Tunability of the bandwidth while keeping the suppression ratio constant. (d) Tunability of the frequency while using an electrical delay to compensate for the limited suppression ratio. (e) Variation of frequency responses for different electrical delays. (f) Tunability of the resonance.

Figure 7(a) presents a preliminary demonstration of the band-stop filters. The bandwidth changed from 1.59 to 5.29 GHz, and the limited suppression ratio ranged from 3.98 to 21.45 dB. As shown in Figure 7(b) and (c), such band-stop filters can tune the suppression ratio while maintaining the bandwidth, vice versa. The change in the matching situation can be compensated for by applying an electrical delay between the two driving RF signals. Figure 7(d) illustrates the tunability of the frequency when using an electrical delay. A suppression ratio above 30 dB could be readily achieved with a maximum of 42.07 dB, whereas the frequency was tunable from 1.25 to 8.04 GHz. There was an optimal electrical delay for achieving the maximum suppression ratio. Figure 7(e) presents the frequency responses of the bandstop filters for different electrical delays. The suppression ratio increased with the electrical delay until the maximum suppression ratio was achieved. Figure 7(a)–(e) demonstrate the tunable frequency range to be 0.25–8.04 GHz. Figure 7(f) illustrates the tunability of resonance, which is calculated based on the EO response data of the child MZM obtained at different bias voltages. The calculation indicates that resonance can be tuned within the range of 0.15–9.75 GHz, fit well with the experimental results. Under high reverse bias voltages, the resonance is significantly reduced as the EO response remains almost constant with respect to the bias voltages.

### 3.2 Filter robustness

The robustness of the RF photonic filters was experimentally investigated by changing the temperature of a thermostatic heater or the optical carrier wavelength. During this process, the other parameters, including the PN junction bias voltages, relative phases of the modulator arms, and optical carrier wavelength/temperature were kept constant.

**Figure 8:**
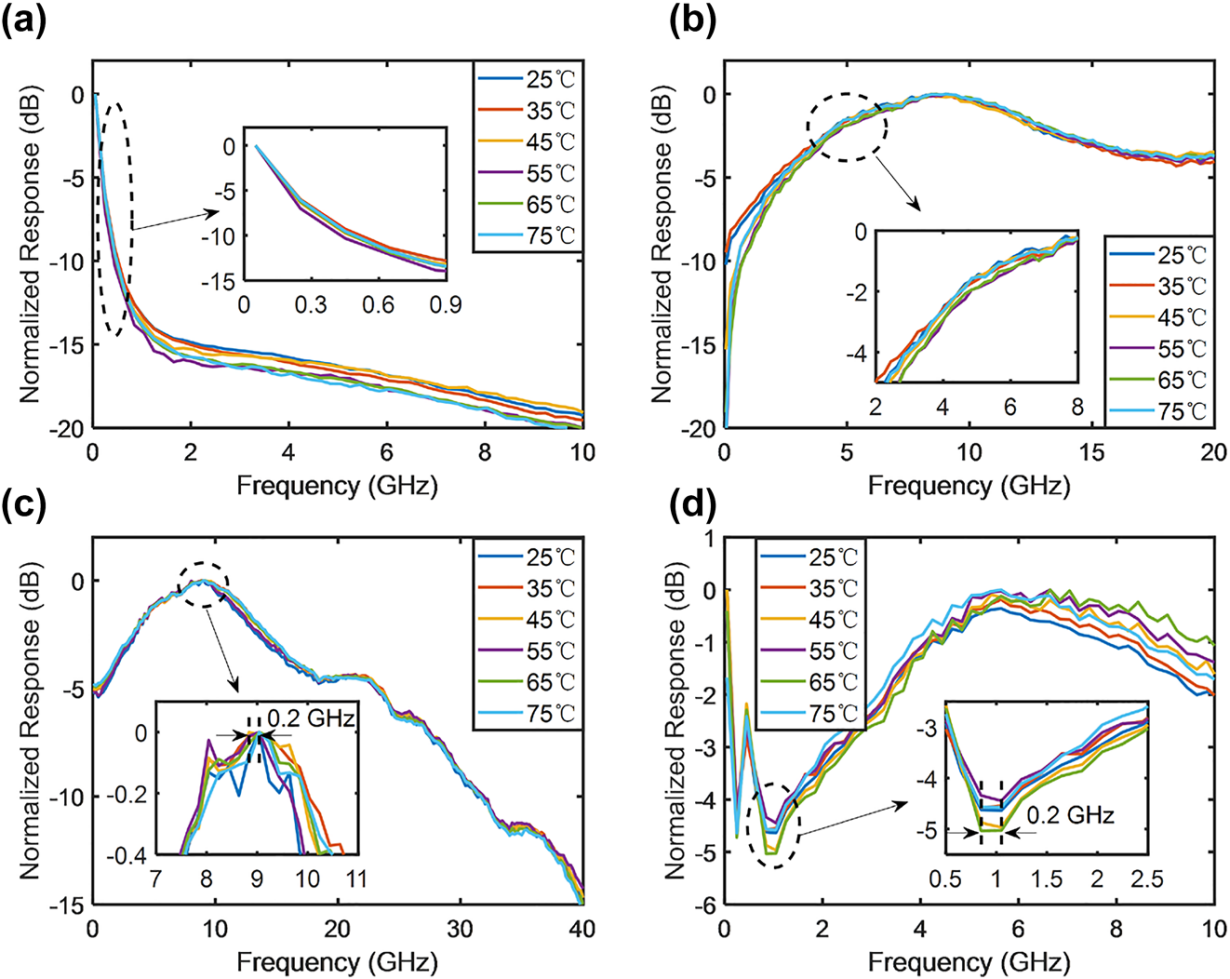
Frequency responses of RF photonic filters for varying temperatures. The temperature varied from 25 to 75 °C in intervals of 10 °C. All frequency responses were normalized to 0 dB. (a) Low-pass filters. (b) High-pass filters. (c) Band-pass filters. (d) Band-stop filters.

**Figure 9:**
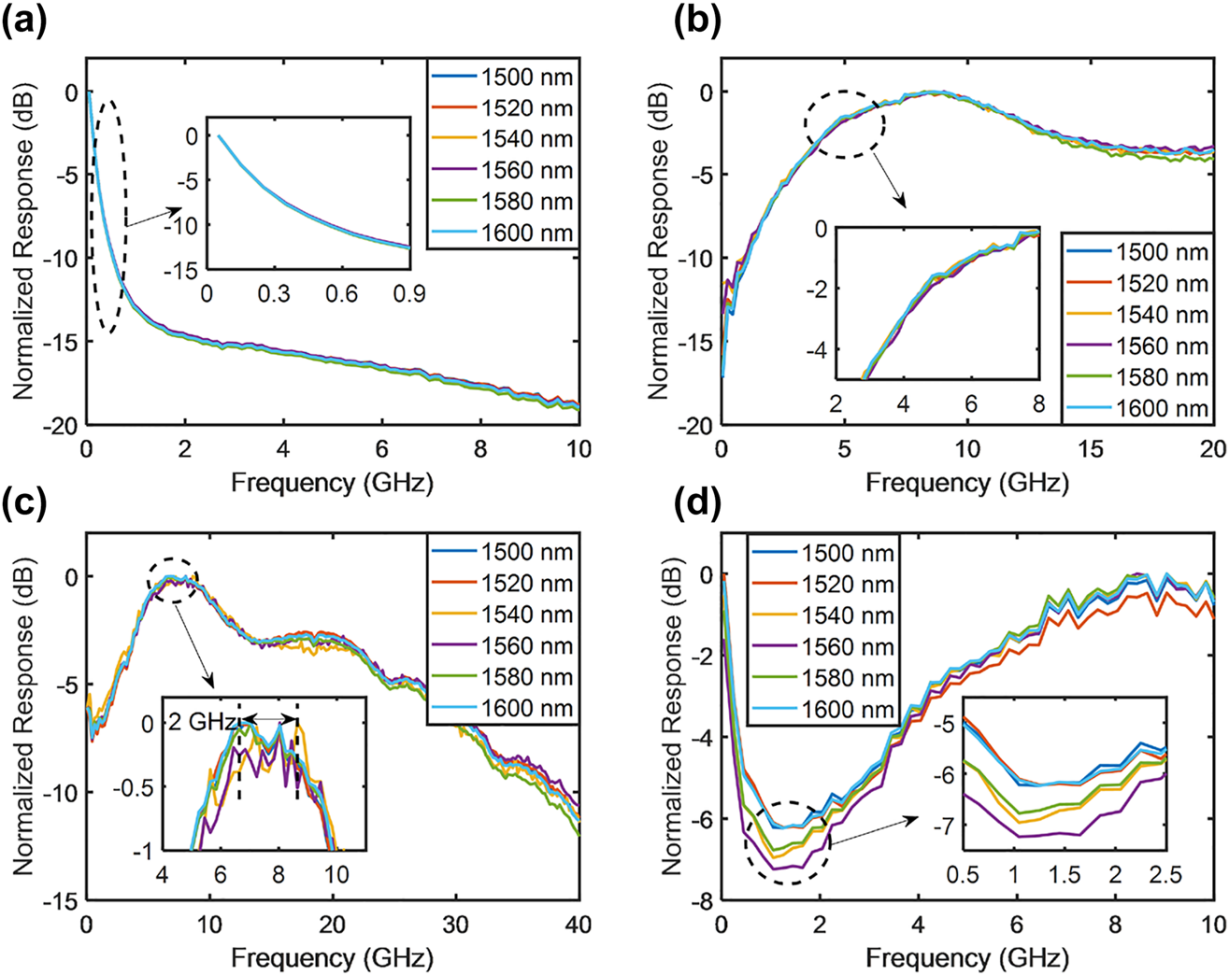
Frequency responses of RF photonic filters for varying optical carrier wavelengths. The optical carrier wavelength varied from 1500 to 1600 nm in intervals of 20 nm. All frequency responses were normalized to 0 dB. (a) Low-pass filters. (b) High-pass filters. (c) Band-pass filters. (d) Band-stop filters.

Figures 8 and 9 illustrate the temperature and optical carrier wavelength experiments of the RF photonic filters, respectively. The temperature was varied from 25 to 75 °C in intervals of 10 °C, and the optical carrier wavelength was varied from 1500 to 1600 nm in intervals of 20 nm. The frequency response exhibited minimal changes during both experiments, thus indicating that the RF photonic filters were highly robust to temperature and optical carrier wavelength variations. The change in the frequency response was caused by the slightly imbalanced arm lengths of the modulator, which were limited by the fabrication process, in addition to the different working effects of thermo-optic tuners at different temperatures and optical carrier wavelengths. The center frequency variation of the RF photonic filters in the temperature and optical carrier wavelength experiments were within a range of 0.2–2 GHz, respectively. Compared with other integrated RF photonic filters based on silicon resonators [18,19,26,27], both the temperature and optical carrier wavelength sensitivities of the IQ modulator-based RF photonic filters were suppressed by more than three orders of magnitude.

## 4 Discussion

Table 1 summarizes the recently reported integrated RF photonic filters. Our technology stands out by employing an IQ modulator, allowing for the implementation of multiple filter types. This provides greater flexibility and functionality compared to other reported filters. However, it is worth noting that the frequency tuning range of our proposed filter is relatively limited due to the constraints imposed by the design of the child MZM. On the other hand, the proposed filter demonstrates a competitive tuning range in terms of bandwidth and rejection ratio when compared to the majority of reported filters. This means that the proposed filter can effectively adjust the bandwidth of the filtered signal and suppress unwanted signals to a significant degree. In previous reports, all the filters utilized optical processing modules, particularly resonant structures such as microring resonators and Bragg gratings. This reliance on optical processing modules has limited the stability of these filters due to temperature drift characteristics of the optical components and wavelength drift of the laser source. However, our technology overcomes these limitations, making it well-suited for applications where temperature and wavelength variations are a concern.

**Table 1:** Comparisons of reported integrated RF photonic filters.

Platform	Technology	Function	Frequency tuning (GHz)	Bandwidth tuning (GHz)	Rejection ratio tuning (dB)	Wavelength sensitivity	Temperature sensitivity
SOI [28]	Photonic crystal	BSF	12.9–32.3	N/A	11.8–62.1	High	High
SOI [18]	Ring resonator	BPF	2–18.4	N/A	N/A	High	High
Si_3_N_4_ [29]	DR-MZI	BPF and BSF	4–25	4.54–9.72	N/A	High	Medium
InP [30]	DBRL	BPF and BSF	9.3–12.3	N/A	N/A	High	High
SOI [31]	Ring resonator	BPF	N/A	5.3–19.5	N/A	High	High
Si_3_N_4_ [10]	Ring resonator	BPF	2–14	0.673–2.798	N/A	High	Medium
InP + SOI [32]	Ring resonator	BPF and BSF	3–25	N/A	N/A	High	High
SOI [33]	MT + ring resonator	BPF and BSF	5–25	N/A	N/A	High	High
Si_3_N_4_ + As_2_S_3_ [34]	SBS + ring resonator	BSF	5–20	N/A	N/A	High	Medium
SOI [35]	ICSSA-CM	BPF and BSF	5–30	0.25–2	12–50	High	High
SOI (this work)	IQ modulator	LPF, HPF, BPF and BSF	0.25–10.04	2.01–19.53	3.98–42.07	Low	Low

LPF, low-pass filter; HPF, high-pass filter; BPF, band-pass filter; BSF, band-stop filter; DR-MZI, dual-ring coupled Mach–Zehnder interferometer; DBRL, distributed Bragg reflection laser; ICSSA-CM, intensity-consistent single-stage-adjustable cascaded-microring; MT, modulation transformer; N/A, not applicated.

## 5 Conclusions

This paper proposes reconfigurable RF photonic filters that can upconvert and process signals on a single silicon IQ modulator. They are robust to temperature and optical carrier wavelength variations, which are difficult to achieve in current integration schemes. Nevertheless, due to the restrictions imposed by the present design, the proposed filter may not perform optimally for certain applications in real-world systems. For example, the limited number of modulation stages used in the RF filter, which comprised only two child MZMs, may account for these limitations. Additionally, it should be noted that the frequency response of the child MZMs can influence the performance of the filter. To address these limitations and enhance the filter performance in future work, multiple potential approaches can be considered. One approach is to increase the number of modulation stages by connecting more child modulators in series or parallel and employing multi-electrode modulators. This architectural expansion is supported by advanced silicon photonic integration technology. Another feasible solution is to improve the shape of the frequency response curve of the modulator by optimizing its design parameters and applying frequency response shaping. By embedding digital signal processing filters in the MZM, the desired frequency response shape can be achieved [36]. Moreover, multiple PN junction bias voltages can be introduced to the frequency response shaping structure to achieve advanced filtering functions within the child MZMs. Overall, considering these improvements, the filter performance can be significantly enhanced for various applications in real-world systems.

In summary, we demonstrated robust reconfigurable RF photonic filters based on a single conventional silicon IQ modulator. In contrast to previous integration schemes that require optical processing modules, we verified that the upconverting and filtering of signals can be simultaneously implemented using a single silicon modulator. The reconfigurability among low-pass, high-pass, band-pass, and band-stop filters, in addition to the tuning of the center frequency and 3 dB bandwidth, can be achieved by appropriately setting the PN junction bias voltages of the two child MZMs and the relative phase of the four modulated arms inside the silicon modulator. The proposed scheme significantly suppresses both the temperature and optical carrier wavelength sensitivities of the RF photonic filters by more than three orders of magnitude. This addresses a significant gap in the literature, given that there is limited research in this area. Additionally, the proposed scheme can serve as a novel approach to signal processing and simplifies the system architecture. By significantly improving the filtering robustness and leveraging mature silicon photonic integration technology, this scheme allows for integrated RF photonic filters to further meet the requirements of practical applications such as modern wireless communications and electronic warfare.

## 6 Methods

### 6.1 Experimental setup

A tunable C-band CW laser was used as the optical carrier source for the optical carrier wavelength tuning experiment. The optical carrier was boosted by an erbium-doped fiber amplifier (EDFA), aligned to the TE mode using a polarization controller (PC), and coupled in and out of the silicon chip using a grating coupler. The fiber-to-chip coupling efficiency was approximately −3.5 dB. A four-port VNA was used to generate two identical RF signals to drive the two child MZMs of the silicon IQ modulator. The RF signals were modulated on the optical carrier and processed by the IQ modulator using high-bandwidth probes. The silicon chip was placed on a thermostatic heater for the temperature tuning experiment. After coupling out the silicon chip, the optical signal was amplified by an additional EDFA and converted to an RF signal using a 50-GHz commercial PD.

### 6.2 Silicon IQ modulator

The silicon IQ modulator used in the experiment was fabricated by AMF, Singapore. The resistivity of the silicon substrate on the silicon-on-insulator (SOI) wafer was 750 Ω cm, and the thicknesses of the silicon and buried oxide layers were 220 nm and 2 μm, respectively. The two parallel child MZMs of the silicon IQ modulator had a mirror-symmetrical structure with an SPP configuration. The parallel connection of the child MZMs was implemented using multimode interferometers (MMIs), and the spacing of the child MZMs was approximately 500 μm. Both the child MZMs and the parent MZM had two arms of equal length. The phase shifter length of the child MZMs was 3.5 mm. Thermo-optic tuners made of TiN were used to set the operating points.

The width and height of the waveguides were 500 nm and 220 nm, respectively. The slab thickness was 90 nm. Two horizontal PN junctions were connected in the SPP mode, which could be biased using a single DC source connected to the heavily-doped region between the two waveguides. The heavily-doped region was separated from the waveguide by a distance of 700 nm to achieve a trade-off between the optical propagation loss and the modulation bandwidth. The traveling-wave electrodes of the child MZMs were made of aluminum metal with a thickness of 2 μm. The traveling-wave electrode was a symmetrical coplanar stripline structure with a width and gap of 40 μm and 9 μm, respectively, which was designed to reduce microwave attenuation, satisfy the characteristic impedance of 50 Ω, and achieve EO group-index matching. An on-chip terminator, which had an impedance of 20 Ω to exploit the bandwidth peaking effect [24], was fabricated at the end of the traveling-wave electrode.

## Supplementary Material

Supplementary Material Details

## References

[1] Capmany J., Ortega B., Pastor D. (2006). A tutorial on microwave photonic filters. *J. Light. Technol.*.

[2] Seeds A. J., Williams K. J. (2006). Microwave photonics. *J. Light. Technol.*.

[3] Capmany J., Novak D. (2007). Microwave photonics combines two worlds. *Nat. Photonics*.

[4] Yao J. (2009). Microwave photonics. *J. Light. Technol.*.

[5] Marpaung D., Roeloffzen C., Heideman R., Leinse A., Sales S., Capmany J. (2013). Integrated microwave photonics. *Laser Photon. Rev.*.

[6] Marpaung D., Yao J., Capmany J. (2019). Integrated microwave photonics. *Nat. Photonics*.

[7] Xu X., Tan M., Wu J. (2019). High performance RF filters via bandwidth scaling with Kerr micro-combs. *APL Photonics*.

[8] Hu J., He J., Liu J. (2020). Reconfigurable radiofrequency filters based on versatile soliton microcombs. *Nat. Commun.*.

[9] Gertler S., Otterstrom N. T., Gehl M. (2022). Narrowband microwave-photonic notch filters using Brillouin-based signal transduction in silicon. *Nat. Commun.*.

[10] Li J., Zheng P., Hu G., Zhang R., Yun B., Cui Y. (2019). Performance improvements of a tunable bandpass microwave photonic filter based on a notch ring resonator using phase modulation with dual optical carriers. *Opt. Express*.

[11] Pan S., Tang Z., Huang M., Li S., Morton P. A., Bowers J. E. (2020). Ring-resonator based widely-tunable narrow-linewidth Si/InP integrated lasers. *IEEE J. Sel. Top. Quant. Electron.*.

[12] Sancho J., Bourderionnet J., Lloret J. (2012). Integrable microwave filter based on a photonic crystal delay line. *Nat. Commun.*.

[13] Marpaung D., Morrison B., Pagani M. (2015). Low-power, chip-based stimulated Brillouin scattering microwave photonic filter with ultrahigh selectivity. *Optica*.

[14] Liu Y., Yu Y., Yuan S., Xu X., Zhang X. (2016). Tunable megahertz bandwidth microwave photonic notch filter based on a silica microsphere cavity. *Opt. Lett.*.

[15] Choo G., Madsen C. K., Palermo S., Entesari K. (2018). Automatic monitor-based tuning of an RF silicon photonic 1×4 asymmetric binary tree true-time-delay beamforming network. *J. Light. Technol.*.

[16] Liu Y., Choudhary A., Marpaung D., Eggleton B. J. (2020). Integrated microwave photonic filters. *Adv. Opt. Photon.*.

[17] Nawrocka M. S., Liu T., Wang X., Panepucci R. R. (2006). Tunable silicon microring resonator with wide free spectral range. *Appl. Phys. Lett.*.

[18] Qiu H., Zhou F., Qie J. (2018). A continuously tunable sub-gigahertz microwave photonic bandpass filter based on an ultra-high-Q silicon microring resonator. *J. Light. Technol.*.

[19] Arbabi A., Goddard L. L. (2013). Measurements of the refractive indices and thermo-optic coefficients of Si_3_N_4_ and SiO_x_ using microring resonances. *Opt. Lett.*.

[20] Lin X., Cheng X., Li F. (2014). Temperature-insensitive laser frequency stabilization to molecular absorption edge using an acousto-optic modulator. *Opt. Lett.*.

[21] Wang H. M., Xu Z. S., Ma S. C., Cai M. H., You S. H., Liu H. P. (2019). Artificial modulation-free Pound–Drever–Hall method for laser frequency stabilization. *Opt. Lett.*.

[22] Li J., Liu Z., Geng Q., Yang S., Chen H., Chen M. (2019). Method for suppressing the frequency drift of integrated microwave photonic filters. *Opt. Express*.

[23] Yu H., Bogaerts W. (2012). An equivalent circuit model of the traveling wave electrode for carrier-depletion-based silicon optical modulators. *J. Light. Technol.*.

[24] Ma Y., Zhang Z., Zhang S. (2019). Self-calibrating microwave characterization of broadband Mach–Zehnder electro-optic modulator employing low-speed photonic down-conversion sampling and low-frequency detection. *J. Light. Technol.*.

[25] Nakamura S., Takahashi S., Ogura I. (2012). High extinction ratio optical switching independently of temperature with silicon photonic 1×8 switch. *Optical Fiber Communication Conference*.

[26] Liu J., Deng H., Zhang W., Yao J. (2018). On-chip sensor for simultaneous temperature and refractive index measurements based on a dual-passband microwave photonic filter. *J. Light. Technol.*.

[27] Zhang W., Yao J. (2018). On-chip silicon photonic integrated frequency-tunable bandpass microwave photonic filter. *Opt. Lett.*.

[28] Long Y., Xia J., Zhang Y., Dong J., Wang J. (2017). Photonic crystal nanocavity assisted rejection ratio tunable notch microwave photonic filter. *Sci. Rep.*.

[29] Yang H., Li J., Zheng P., Hu G., Yun B., Cui Y. (2019). A stopband and passband switchable microwave photonic filter based on integrated dual ring coupled Mach–Zehnder interferometer. *IEEE Photon. J.*.

[30] Zou X., Zou F., Cao Z. (2019). A multifunctional photonic integrated circuit for diverse microwave signal generation, transmission, and processing. *Laser Photon. Rev.*.

[31] Xu L., Hou J., Tang H. (2019). Silicon-on-insulator-based microwave photonic filter with widely adjustable bandwidth. *Photon. Res.*.

[32] Tao Y., Shu H., Wang X. (2021). Hybrid-integrated high-performance microwave photonic filter with switchable response. *Photon. Res.*.

[33] Guo X., Liu Y., Yin T. (2021). Versatile silicon microwave photonic spectral shaper. *APL Photonics*.

[34] Garrett M., Liu Y., Merklein M. (2022). Multi-band and frequency-agile chip-based RF photonic filter for ultra-deep interference rejection. *J. Light. Technol.*.

[35] Tao Z., Tao Y., Jin M. (2023). Highly reconfigurable silicon integrated microwave photonic filter towards next-generation wireless communication. *Photon. Res.*.

[36] Breyne L., Lambrecht J., Verplaetse M. (2021). Electro-optic frequency response shaping using embedded FIR filters in slow-wave modulators. *J. Light. Technol.*.

