# Supplementary material for: Robust reconfigurable radiofrequency photonic filters based on a single silicon in-phase/quadrature modulator
Source: Nanophotonics. 2023 Oct 25;12(22):4175–84. doi: 10.1515/nanoph-2023-0459 (PMC11501990; doi:10.1515/nanoph-2023-0459)
Supplement: Supplementary file 1 — Supplementary Material Details [file j_nanoph-2023-0459_suppl_001.docx]

Supplementary Material

Robust reconfigurable radiofrequency photonic filters based on a single silicon in-phase/quadrature modulator

Hengsong Yue and Tao Chu*

College of Information Science and Electronic Engineering, Zhejiang University, Hangzhou 310027, China

*chutao@zju.edu.cn

I. Detailed derivation of the equations provided.

Here, we illustrate the detailed derivation of the equations provided. The complex amplitude of the input optical carrier to the modulator can be expressed as follows:

$\widetilde{E}_{\text{in}}\text{(}t\text{)}=E_{0}\text{exp(}j\omega_{\text{c}}t\text{)}$ （S1）

where *E_0_* and *ω*_c_ are the amplitude and angular frequency of the optical carrier, respectively. The input optical carrier passes through a beam splitter and then enters the two child MZMs. Assuming that the amplitudes of the optical carriers on these two child MZMs are equal, denoted as *E_1_*. Under ideal conditions, we have *E_1_* = (0.5) ^1/2^ *E_0_*. Similarly, the optical carrier from each child MZM passes through a beam splitter and then enters two modulation arms. On the modulation arms, the optical carrier undergoes phase modulation. Assuming that the amplitudes of the optical carriers on these two modulation arms are equal, denoted as *E_2_*. Under ideal conditions, we have *E_2_* = (0.5) ^1/2^ *E_1_* = 0.5 *E_0_*.

Assuming small-signal modulation, the two identical RF signals used to drive the two child MZMs can be denoted by *V*_RF_sin(*ω*_RF_*t*), where *V*_RF_ and *ω*_RF_ are the magnitude and angular frequency of the RF signal, respectively. Due to operating in a series push-pull configuration, the phase variations caused by modulation on the two modulation arms can be expressed as *m*sin(*ω*_RF_*t*) and *m*sin(*ω*_RF_*t*+π) respectively. Assuming *V*_π_ is the half-wave voltage, m = π*V*_RF_/2*V*_π_ is the modulation indice of the child MZM. Hence, for a single child MZM, the complex amplitude expression of the output optical carrier from the two modulation arms can be given as:

$\widetilde{E}_{A}=\frac{E_{0}\text{exp}(j\omega_{\text{c}}t\text{)}}{2}\text{exp}\left[jm\text{sin}(\omega_{\text{RF}}t)\right]$ （S2）

$\widetilde{E}_{B}=\frac{E_{0}\text{exp}(j\omega_{\text{c}}t\text{)}}{2}\text{exp}\left[jm\text{sin}(\omega_{\text{RF}}t+\pi)\right]$ （S3）

The function of the form exp[*jm*sin(*ωt*+*φ*)] can be expanded using Bessel functions:

 （S4）

where *J_n_* is the nth-order Bessel function of the first kind.

Due to the assumption of small signal modulation, higher-order sidebands beyond the first order are neglected. The complex amplitude expression of the output optical carrier from the two modulation arms can be given as:

$\widetilde{E}_{A}=\frac{E_{0}\text{exp}(j\omega_{\text{c}}t\text{)}}{2}\left\{\begin{matrix} -J_{1}\text{(}m\text{)exp}(-j\omega_{\text{RF}}t\text{)} \\ +J_{0}\text{(}m\text{)} \\ +J_{1}\text{(}m\text{)exp}(j\omega_{\text{RF}}t) \end{matrix}\right\}$ （S5）

$\widetilde{E}_{B}=\frac{E_{0}\text{exp}(j\omega_{\text{c}}t\text{)}}{2}\left\{\begin{matrix} J_{1}\text{(}m\text{)exp}(-j\omega_{\text{RF}}t) \\ +J_{0}\text{(}m\text{)} \\ -J_{1}\text{(}m\text{)exp}(j\omega_{\text{RF}}t) \end{matrix}\right\}$ （S6）

The output optical carrier from the child MZM is formed by the interference of the optical carriers from the two modulation arms. The output of the child MZM can be expressed as:

 （S7）

where *φ* is the phase difference of the two arms of the child MZM. Suppose *m*_1_ and *m*_2_ represent the modulation indices of the two child MZMs, while *φ*_1_ and *φ*_2_ denote the phase differences of the two arms of the two child MZMs. The specific representation of the outputs of the two child MZMs can be expressed as follows:

 （S8）

 （S9）

The output of the silicon IQ modulator can be expressed as follows:

 （S10）

where *φ*_3_ is the phase difference between the two arms of the parent MZM. The given expression corresponds to Equation (1) in the research paper.

The output of the silicon IQ modulator was injected into a PD. Neglecting the frequency-doubling and direct current (DC) terms, the photocurrent can be expressed as follows:

 （S11）

where the zeroth order Bessel function of the first kind is set to 1 because small-signal modulation is assumed. The given expression corresponds to Equation (2) in the research paper.
